# Editorial: Long-term toxicity and epigenetic effects of environmental exposures

**DOI:** 10.3389/fgene.2022.1044589

**Published:** 2022-09-30

**Authors:** Douglas M. Ruden, Kai Wang, Kangxu Wang, Bambarendage P. U. Perera, Rebekah Lee Petroff

**Affiliations:** ^1^ Institute of Environmental Health Sciences, C. S. Mott Center for Human Health and Development, Department of Obstetrics and Gynecology, Wayne State University, Detroit, MI, United States; ^2^ Department of Computational Medicine and Bioinformatics, School of Medicine, University of Michigan, Ann Arbor, MI, United States; ^3^ Department of Environmental Health Sciences, School of Public Health, University of Michigan, Ann Arbor, MI, United States; ^4^ Department of Animal Science, College of Agriculture & Natural Resources, Michigan State University, East Lansing, MI, United States

**Keywords:** epigenetics, toxicology, transgenerational, DOHAD, toxicoepigenetics, DNA methylation, histone modifications, non-coding RNA

## Introduction

The Developmental Origins of Health and Disease (DOHaD) hypothesis emphasizes how effects of prenatal or perinatal environmental exposures can determine later-life human health and disease (Suzuki, 2018). While this concept is supported by a variety of experimental and epidemiological studies, explicit mechanisms driving the long-term effects of early-life exposures are not well described. Epigenetic changes, such as differences in DNA methylation, histone modifications, and changes of non-coding RNAs, are suggested as potential regulators of environmentally-induced long-term health effects (Bollati and Baccarelli, 2010). As evidenced in the results of multiple studies, epigenetic modifications can be affected by environmental exposures early in life that impact later health (Perera et al., 2020). For instance, exposure to lead (Pb) in early development can cause the overexpression of genes related to neurodegenerative diseases, resulting in epigenetic changes later in life (Dórea, 2019).

With recent technological advancements in genomic research, several next-generation sequencing-based omics techniques can be used to detect genome-wide epigenetic changes. These techniques include chromatin immunoprecipitation (ChIP-seq) (Park, 2009) and DNase I hypersensitive sites-sequencing (DNase1-seq) (Furey, 2012) for detecting DNA-protein interactions; assay for transposase-accessible chromatin using sequencing (ATAC-seq) (Buenrostro et al., 2015) for discovering chromatin accessibility; chromatin isolation by RNA purification (ChiRP-seq) (Chu et al., 2012) for identifying DNA-RNA interactions; and whole genome bisulfite sequencing (WGBS) (Suzuki et al., 2018) and enhanced reduced representation bisulfite sequencing (ERRBS) (Garrett-Bakelman et al., 2015) for distinguishing DNA methylation changes. Applying these techniques, one can detect and characterize the epigenetic changes caused by environmental exposures and discover potential molecular mechanisms involved in regulating gene expression that cause adverse health effects in later life.

Three of the studies, using children from the ECHO (Gillman and Blaisdell, 2018) longitudinal cohort of over 50,000 children in the United States, investigated how prenatal exposures to environmental chemicals affect DNA methylation in the children: Petroff et al. showed how phthalates and common phthalate replacement chemicals are associated with DNA methylation in newborns in a sex-specific manner (https://www.frontiersin.org/articles/10.3389/fgene.2022.793278/full); Guo et al. developed statistical approaches that correlated neonatal exposures including phenols, pesticides, phthalates, flame retardants, and air pollutants with DNA methylation changes in children and their corresponding cognitive, behavioral, and mental health outcomes (https://www.frontiersin.org/articles/10.3389/fgene.2022.871820/full); and Song et al. showed how prenatal ambient air pollution exposures during pregnancy are associated with decelerated epigenetic aging in the newborns (https://www.frontiersin.org/articles/10.3389/fgene.2022.929416/full).

In an EWAS using umbilical cord blood from a seperate cohort, Ulloa et al. identified several genes that have an altered DNA methylation pattern in children exposed to 2–8.5 μg/L MeHg and confirmed these DNA methylation changes in several of the genes by exposing SH-SY5Y neuroblastoma cells to 8 or 40 nM MeHg (https://www.frontiersin.org/articles/10.3389/fgene.2022.993387/abstract). In a human study with adult men, Maggioet al. showed how elevated exposures to persistent endocrine disrupting compounds can impact the sperm methylome in regions associated with autism spectrum disorder (https://www.frontiersin.org/articles/10.3389/fgene.2022.929471/full). Finally, two studies investigated differences in microRNAs with environmental exposures, in non-human models: Yang et al. showed how four differentially expressed microRNAs mediate insecticide tolerance in *Spodoptera frugiperda* (https://www.frontiersin.org/articles/10.3389/fgene.2021.820778/full); and Zhang et al. conducted miRNA expression analysis in *Bemisia tabaci* under insecticide tolerance to assess stable reference genes for qPCR analyses (https://www.frontiersin.org/articles/10.3389/fgene.2022.899756/full). These studies contribute to the burgeoning progress in understanding mechanisms of long-term toxicity and epigenetic effects upon environmental exposures.
